# Differential Involvement of the Anterior Temporal Lobes in Famous People Semantics

**DOI:** 10.3389/fpsyg.2016.01333

**Published:** 2016-08-30

**Authors:** Georges Chedid, Maximiliano A. Wilson, Jean-Sebastien Provost, Sven Joubert, Isabelle Rouleau, Simona M. Brambati

**Affiliations:** ^1^Centre de Recherche de l'Institut Universitaire Gériatrique de MontréalMontréal, QC, Canada; ^2^Department of Psychology, Université de MontréalMontréal, QC, Canada; ^3^Centre de Recherche de l'Institut Universitaire en Santé Mentale de QuébecQuébec, QC, Canada; ^4^Department of Psychology, Université du Québec à MontréalMontréal, QC, Canada

**Keywords:** fMRI, semantic memory, anterior temporal lobe, famous faces, famous names

## Abstract

The ability to recognize a famous person occurs through semantic memory. Previous neuroimaging studies have shown that the anterior temporal lobes (ATLs) are involved in the recognition of famous people. However, it is still a matter of debate whether the semantic processing of names or pictures of famous people has an impact on the activation of ATLs. The aim of this study was to explore the pattern of activation associated with a semantic processing of famous people based on face and written name stimuli. Fifteen healthy young individuals participated in our fMRI study, in which they were asked to perform a semantic categorization judgment task, based on profession, of visually presented pictures, and names of famous people. Neuroimaging findings showed a common pattern of activation for faces and names mainly involving the inferior frontal regions, the posterior temporal lobe, the visual cortex, and the ATLs. We found that the comparison names vs. pictures lead to significant activation in the anterior superior temporal gyrus. On the other hand, faces vs. names seemed associated with increased activation in the medial ATL. Moreover, our results demonstrated that the functional connectivity network anchored to the medial ATL, compared to the anterior STG, is more connected to the bilateral occipital lobe and fusiform gyrus that are regions implicated in the visual system and visual processing of faces. This study provides critical evidence of the differential involvement of ATL regions in semantics of famous people.

## Introduction

Semantic memory is part of the long-term memory system where the conceptual knowledge of people, objects, sounds, and words is stored (Levy et al., 2004). However, there is converging evidence on the central role of the anterior temporal lobe (ATL) within the semantic memory system. Evidence mainly comes from patients with the semantic variant of primary progressive aphasia (svPPA), also referred to as semantic dementia (SD; Hodges et al., 1992; Neary et al., 1998; Gorno-Tempini et al., 2011). These patients are characterized by isolated progressive semantic memory loss (Hodges et al., 1992; Patterson et al., 2007), which is associated with a bilateral but asymmetrical atrophy of the lateral ATLs. These patients present multi-modal semantic deficits in the context of relatively spared abilities in other cognitive domains such as episodic memory and short-term memory. Secondly, studies using transcranial magnetic stimulation (TMS) have shown that stimulation interfering with the proper functioning of the ATLs is associated with decreased performance in semantic tasks, inducing semantic deficits similar to those observed in svPPA patients, but not in other equally demanding non-semantic cognitive tasks (Pobric et al., 2007). However, functional neuroimaging studies on healthy individuals using positron emission tomography (PET) and functional magnetic resonance imaging (fMRI) have often provided inconsistent results on the implication of ATLs in semantic tasks. This is mainly due to the fact that fMRI presents magnetic susceptibility affecting the blood oxygenation level dependent (BOLD) signal in the ATL region (Devlin et al., 2000), especially in the ventral areas (Visser et al., 2010). Visser et al. (2010) conducted a meta-analysis based on 164 PET and fMRI studies on semantic processing in order to identify factors that could have contributed to these inconsistencies. Their meta-analysis revealed four different factors that could increase the likelihood of detecting significant activation of the ATLs in association with a semantic task: (1) the use of PET vs. fMRI acquisition, since PET is less sensitive to signal distortions in the ATLs, (2) the use of a field of view (FOV) to ensure whole-brain coverage; (3) the use of high baseline task (vs. “rest”), and (4) the inclusion of ATL as a region of interest (ROI). However, the meta-analysis indicated that the type of stimuli did not have an impact of the likelihood of ATL activation. Altogether, these findings seem to indicate that the ATLs could have a transmodal role within the semantic system.

Recently, Rogers et al. (2004) proposed a “hub-and-spoke” model of conceptual representation within the brain (Rogers et al., 2004; Lambon Ralph, 2014). According to this model, the representation of conceptual knowledge activates the ATL system that is bilateral, transmodal and connected to various modality-specific sensory, motor, and limbic cortices. This model combines the “distributed-only” theory, that states that the information coming from a specific modality is stored in a specific cortical sensory, motor or language area (the “spokes”), with the “distributed-plus-hub” theory, which proposes a neuroanatomical pathway between different sensory, motor and linguistic regions that converge to a transmodal hub supporting the interactive activation of representations in all modalities. Within this framework, the ventrolateral ATL would represent the key region of the hub (Lambon Ralph et al., 2001; Rice et al., 2015). Evidence comes from different lines of research. A multi-voxel pattern analysis fMRI study linked the contribution of the ventrolateral ATL during semantic processing with delivering information that is independent of the perceptual properties of the stimulus (i.e., about how and where an object is typically used; Peelen and Caramazza, 2012). Secondly, studies using subdural electrode grids over the ventral ATL subregions confirmed its contribution in both expressive (picture naming) and receptive semantic tasks (synonym judgments; Shimotake et al., 2014). Thirdly, the stimulation of this area via TMS has led to transiently impaired performance in semantic tasks with both visual and auditory inputs (i.e., picture naming, spoken and written word-picture matching, etc.). Lastly, according to a meta-analysis of 97 fMRI studies, the ventrolateral portions of the ATLs were shown to receive converging inputs from the primary sensory cortices and medial temporal structures (Rice et al., 2015).

However, some regions of the ATL seem to show differential specialization for verbal vs. visual processing. Some studies have proposed that this specialization would depend on the differential contribution of the left and right hemisphere. Studies assessing semantic knowledge in patients with svPPA have shown that individuals with greater atrophy of the left ATL performed more poorly with written word stimuli compared to faces (Snowden et al., 2004, 2012). Conversely, patients with greater atrophy of the right ATL showed a reverse pattern. These results may suggest a differential involvement of the left and right ATLs depending on the modality of stimulus presentation. This effect could be determined by the dominance of the left hemisphere for language and of the right hemisphere for the perceptual processing of images. However, this hypothesis does not seem to be supported by the results of a previous TMS study on healthy participants (Pobric et al., 2010). The study revealed comparable selective impairments in the semantic processing of words and pictures, which was caused by a temporary lesion produced over either the left or the right temporal poles. In another account, other studies have shown a differential involvement of different regions within the ATLs that are stronger than any left vs. right laterality effects. More specifically, the superior ATL has shown greater activation for auditory and verbal stimuli compared to pictures (Moore and Price, 1999; Visser and Lambon Ralph, 2011; Visser et al., 2012), while ventromedial ATL has shown greater activity for pictures relative to words (Visser et al., 2012). It has been proposed that this differential specialization of different regions of the ATL would be due to the different strengths of connection between theses portions of the ATL and modality-specific regions, namely the posterior superior temporal gyrus for words (auditory language network) and the ventral posterior temporal cortex for images (visuo-perceptual network; Lambon Ralph et al., 2001; Rice et al., 2014). In fact, based on semantic models and neuroimaging studies, the ATL as a whole is connected to an anatomically distributed semantic network (Patterson et al., 2007), but also, there are regions within the ATL that may have preferential connectivity with graded strength (Rice et al., 2015) to specific temporal, frontal and parietal areas (Visser and Lambon Ralph, 2011; Visser et al., 2012).

While the differential implication of different ATL regions has been consistently reported for objects, the effect of input modality of the semantic processing of known people is less clear. The aim of the present study were: (1) to identify the common pattern of activation associated to the semantic processing of famous faces and famous names; (2) to verify whether the activation of the ATLs is modulated by the modality of the stimulus presentation (names vs. pictures); (3) to test whether the regions of ATL differently implicated in the semantic processing of faces and names show differences in the pattern of functional connectivity with the visuo-perceptual or language-related brain regions. For this purpose, an fMRI study was performed with a group of healthy young adults engaged in a semantic categorization task of famous people based on profession. For fMRI data acquisition, field of view was set in a way that ensures whole-brain coverage (see **Figure 2**).

## Materials and methods

### Participants

A group of 15 healthy young adults (8 males and 7 females), between 20 and 33 years old (mean age = 25.4 years; *SD* = 4.12), participated in the present study. Participants were all native French speakers and right-handed, as assessed by the Edinburgh Handedness Inventory (Oldfield, 1971). All participants provided their informed, written consent, and this study was reviewed and approved by the local ethics committee at the *Institut Universitaire de Gériatrie de Montréal* (IUGM) and the *Comité Mixte D'éthique de la Recherche du Regroupement Neuroimagerie Québec* (CMER-RNQ). This committee follows the guidelines of the Tri-Council Policy Statement of Canada, the civil code of Quebec, the Declaration of Helsinki, and the code of Nuremberg.

### Stimuli selection

The stimuli were selected based on a pilot study carried out with a group of 10 young healthy French-speaking individuals, between 18 and 30 years old. Participants were asked to spontaneously produce 40 names of actors/actresses and 40 singers. A list including the names of the most cited actors/actresses (*n* = 21) and singers (*n* = 20) was then produced. A separate group of volunteers was then invited to perform a familiarity judgment task on the produced list of names in which they had to rate each item on how well they felt they knew each artist on a 7-point Likert scale, in which “1” corresponded to very little known and “7” to very well-known. Participants were explicitly told to use all the numbers of the scale and to rate all of the stimuli. In order to include only highly familiar items in the study, we only retained the artists presenting a mean familiarity cutoff of 5.5 or higher. The 15 most familiar actors/actresses (mean familiarity = 6.20 ± 0.57; *n* = 15) and singers (mean familiarity = 6.29 ± 0.52; *n* = 15) were selected and included in the fMRI task. No significant difference was observed in terms of familiarity between actors/actresses and singers. In order to verify whether the participants could recognize the names and faces of the chosen items, we selected color pictures of the faces of the 30 selected items. The background was removed from all pictures. A group of seven volunteers (who had not participated in the previous pilot phase) was invited to identify the name and profession of each famous face. All of the faces were correctly identified and categorized based on profession in at least 75% of the cases. For the control condition, a series of unfamous faces was selected. Unfamous faces (UF) were matched with famous faces (FF) according to age, sex, the presence of certain physical attributes, such as glasses or facial hair, and according to brightness. The faces were surrounded by a black oval mask to avoid substantive differences (Gorno-Tempini and Price, 2001).

The unfamous names (UNs) were obtained by combining common first names and last names. The UNs were paired by the number of letters, initial letter of the name and last name, and the origin of the names and last names. A pilot study was then performed with seven volunteers to verify that the names did not remind them of any famous name.

### Experimental design

**Figure 1:**
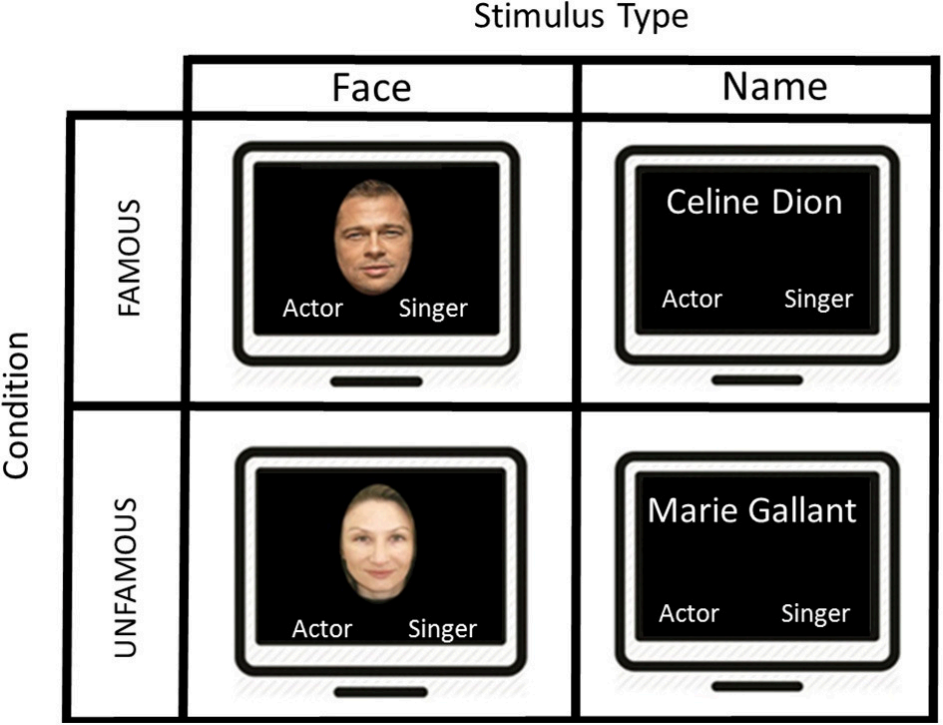
**A schematic illustration of the presentation of the stimuli used in this block design task**. The first screen shows an example of a famous face “Brad Pitt” during 2500 ms and the participant had to make a category profession choice by selecting “actor” or “singer.” An interval of 1500 ms separates the display from the next stimulus. After 5 famous faces, we presented another type of stimuli showing 5 consecutive famous names, and the participant had to judge once again the proper category. Two other conditions were displayed (i.e., unfamous faces and unfamous names), but not showed in this figure.

In the present fMRI study, we used a block design protocol in order to map the pattern of activation associated with the semantic categorization for famous faces and names based on profession. Participants were exposed simultaneously to the target stimulus in the upper center part of the screen and the semantic category label “singer” or “actor” respectively, in the lower right and left part of the screen. The position of the semantic category labels in the left and right part of the screen was counterbalanced across subjects. The images were projected onto a mirror placed in front of the participant in the scanner via an LCD screen (Figure 1).

Four different types of target stimuli were included in the study: (1) Famous Faces (FF condition); (2) Famous Names (FN condition), (3) Unfamous Faces (UF condition), (4) Unfamous Names (UN condition). Our experimental condition included FF and FN, while UF and UN were part of our control conditions.

In the experimental conditions (i.e., FF and FN), participants were instructed to determine as quickly and as accurately as possible whether the target stimulus was an actor or a singer. Unlike previous studies, we employed an explicit semantic categorization task. To provide their answer, a magnetic resonance compatible response box was placed on the participant's abdomen. The left button was associated with the left index finger and corresponded to the “singer” category, and the right button was assigned to the right index finger and was associated with the “actor” category. The response box was connected to a laptop outside the MR room in order to record reaction times (RTs) and response accuracy (Figure 1).

Since the control condition included unfamous items (i.e., UF and UN), we could not use a comparable task to the experimental condition (i.e., semantic categorization task). For this reason, we asked our participants to observe the unknown faces or names, and randomly press on either the right or the left button. This control condition was chosen to subtract the activation associated with the visual processing of UFs or UNs, the reading of category labels and the motor response.

The fMRI experiment was conceived in a block design. Each block consisted of five stimuli displayed for 2500 ms each, followed by a 1500 ms inter-stimulus interval in which a crosshair was displayed on the screen. Each block was interleaved with a rest period, in which a black screen appeared for 20 s. The same items were used for both the FF and FN conditions; the same actor/singer was included as a stimulus in the face and name category. The order of presentation was counterbalanced so that half of the participants were exposed to the name condition first and the other half to the picture condition first. The study included two functional runs. In each run, subjects performed three blocks of each condition viewing 15 FF, 15 UF, 15 FN, and 15 UN.

Participants were asked to respond as quickly and as accurately as possible during the presentation of the stimulus. Any response beyond the presentation time (i.e., 2500 ms) was considered an error. Prior to the scanning session, participants were instructed to arbitrarily respond when they could not recognize the face or name.

### Image acquisition

The acquisition of MRI images was obtained using a 3T Siemens Trio Magnetom scanner (Siemens, Erlangen, Germany) at the Functional Neuroimaging Unit of the *Centre de Recherche de l'Institut Universitaire de Gériatrie de Montréal*. Each subject's head was thoroughly padded in the coil to reduce head motion.

For each run, T2^*^-weighted functional images were acquired using an EPI pulse sequence, in an axial plane aligned with the hippocampus, using the following parameters: TE/TR = 30/4010 ms, Flip angle = 90°, Matrix = 128 × 128 mm, voxel size = 2 × 2 × 2 mm^3^, slice gap = 0.5 mm, 45 slices in an interleaved acquisition. A total of 2 functional acquisitions of about 7 min each were acquired. A volumetric magnetization prepared rapid gradient echo (MPRAGE) sequence was then used to acquire a high-resolution T1-weighted 3D anatomical image, using the following parameters: TR = 2.3 s, TE = 2.91 ms, TI = 900 ms, flip angle = 9°, FOV = 240 × 256, voxel size = 1 × 1 × 1.2 mm^3^. Although this acquisition protocol cannot be considered the optimal protocol to obtain BOLD signal in the ATL (Poser et al., 2006; Poser and Norris, 2007, 2009; Halai et al., 2014; Jackson et al., 2015), we used acquisition parameters that ensure whole-brain coverage (Visser et al., 2010) and that have been successfully employed to detect significant BOLD signal activation in previous studies by our group (Brambati et al., 2012; Wilson et al., 2012).

### Data analysis

Functional volumes were preprocessed and analyzed using SPM12 implemented in MATLAB. Time-series were corrected and realigned using rigid body transformations for motion correction between volumes and for the reduction of the residual sum of squares between each subsequent image. All volumes were realigned from the estimated mean image. In addition, the T1 acquisition was also realigned. Co-registration parameters were then applied to the realigned BOLD time series. Afterwards, the mapping from the subject to the MNI brain template (i.e., ICBM152) was estimated from the structural image with the standard segmentation approach. Parameter files for spatial normalization were then applied to the individually co-registered BOLD times series, which were spatially smoothed using a Gaussian kernel of FWHM = 8 mm.

The analysis of fMRI data was conducted in two serial steps, accounting respectively for fixed and random effects. For each subject, changes in brain regional responses were estimated through a model including mean RTs for each block as a covariate in the model. This regressor consisted of box cars convolved with the canonical hemodynamic response function. This was done in order to control for the fact that shorter RTs would be associated with more retrieval of post-recognition associations (films the actor was in, songs the singer performed, etc.), thus influencing brain activation. One should note that a limitation of such design could have lower sensitivity for detecting condition effects for items with systematically longer RT.

High-pass filtering was implemented in the design matrix using a cut-off period of 128 s to remove slow drifts from the time series. Serial correlations in fMRI signal were estimated using an autoregressive (order 1) plus white noise model and a restricted maximum likelihood (ReML) algorithm.

These linear contrasts generated statistical parametric maps. The resulting contrast images were then entered in a second level analysis, corresponding to a random effects model, and accounting for inter-subject variance. A level of significance of *p* < 0.05 FWE (SPM family-wise error) corrected at a cluster level was accepted. Based on previous studies, a less conservative threshold of *p* < 0.001 uncorrected for multiple comparisons was adopted for the ATLs.

### Functional connectivity MRI

Functional connectivity MRI is an fMRI method that examines the connectivity of different brain areas based on the correlation of BOLD signal fluctuations over time. Regions of the ATL showing preferential involvement for images or names were used as seed regions for the functional connectivity analysis. We then performed a functional connectivity analysis aiming at identifying inter-regional relationships (Friston, 1994). We calculated correlations between the BOLD timeseries from our regions of interest (ROIs) and the voxels in the brain.

## Results

### Behavioral results

We used the linear mixed effects modeling approach, a type of analysis that controls for the crossed random effects of participants and items (Baayen et al., 2008) in SPSS 24. Task condition (Famous, Unfamous) and modality input (Face, Name) were entered in the model as fixed effects, and reaction times (RTs) and accuracy (Acc) were entered as dependent variables. Missing responses and errors were removed from the analysis. Tables 1, 2 show the mixed model analysis estimates and tests of fixed effects for RTs and Accuracy respectively. Both task condition and modality input significantly affected the RTs. Participants were noticeably faster in performing the semantic categorization task for the famous task condition compared to unfamous condition (mean RTs Famous = 1240.95, SE = 70.21; Unfamous = 1316.35, SE = 70.21), and they were faster when the modality input was Name compared to Face (Name = 1225.09, SE = 70.22; Face = 1332.21, SE = 70.20). Also, the interaction task condition by input modality significantly affected RTs. Simple effects analyses by task condition showed that participants were faster responding to names than faces when the condition was famous (FN = 1137.59 ms, SE = 71.39; FF = 1344.32 ms, SE = 7139), which was not the case when the condition was unfamous (UN = 1312.60 ms, SE = 71.43; UF = 1320.10 ms, SE = 71.36). There was no significant effect of the modality during unfamous condition on RTs. Table 3 shows the mixed model analysis estimates and tests of fixed effects for the interaction task condition by input modality.

**Table 1:** **Mixed model analysis estimates for condition and modality for RTs**.

**Source**	**Numerator**	**Denominator**	***F***	**Sig**.
	***df***	***df***		
Intercept	1	19.56	337.41	0.000^*^
Condition	1	1712.70	16.96	0.000^*^
Modality	1	1712.69	34.22	0.000^*^
Condition × Modality	1	1712.68	29.59	0.000^*^

*Dependent variable: RTs*.

* *p < 0.001*.

**Table 2:** **Mixed model analysis estimates for condition and modality for accuracy**.

**Source**	**Numerator**	**Denominator**	***F***	**Sig**.
	***df***	***df***		
Intercept	1	29.70	12,003.63	0.000^*^
Condition	1	1713.52	57.51	0.000^*^
Modality	1	1713.57	1.64	0.200
Condition × Modality	1	1713.87	0.99	0.319

*Dependent variable: Accuracy*.

* *p < 0.001*.

**Table 3:** **Mixed model analysis estimates for the interaction modality by condition for RTs, as a function of condition**.

**Condition**		**Numerator**	**Denominator**	***F***	**Sig**.
		***df***	***df***		
Famous	Intercept	1	17.87	621.69	0.000^*^
	Modality	1	836.34	79.22	0.000^*^
Unfamous	Intercept	1	23.84	151.79	0.000^*^
	Modality	1	833.96	0.11	0.734

*Dependent variable: RTs*.

* *p < 0.001*.

The analysis of accuracy revealed a significant effect for task condition. Participants were more accurate for the unfamous condition than the famous one (mean accuracy Unfamous = 0.99, SE = 0.01; Famous = 0.94, SE = 0.01). Input modality (Names = 0.97, SE = 0.01; Face = 0.96, SE = 0.01) or the interaction task condition by input modality did not significantly affect accuracy (Table 2).

### fMRI results

**Figure 2:**
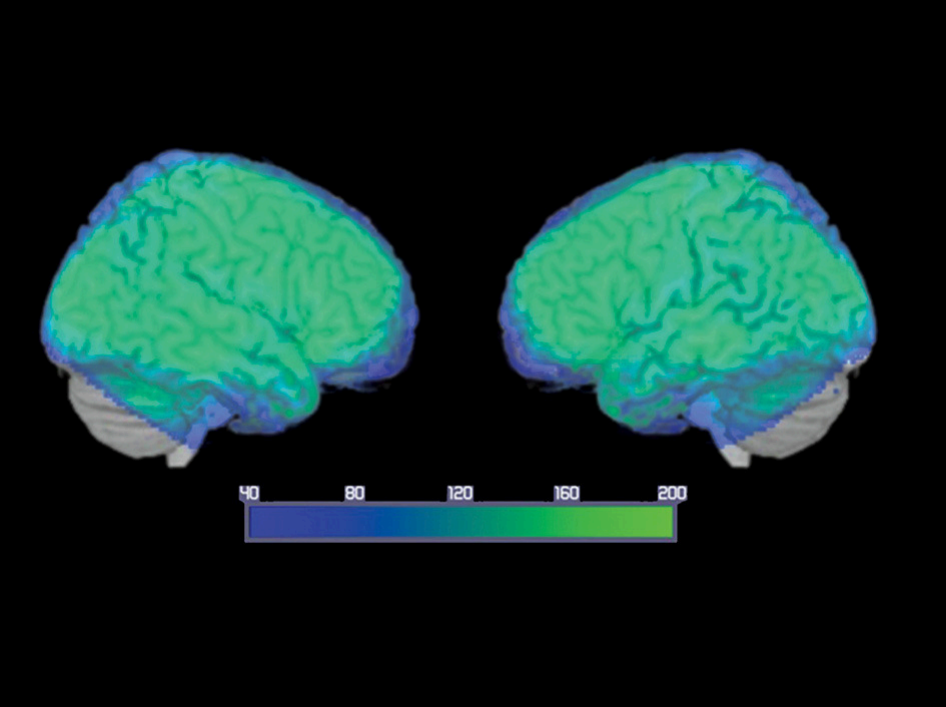
**Average temporal signal-to-noise ratio (tSNR) maps, for the smoothed group echo planar imaging data in MNI space, showing EPI image quality over the ATLs**. The color map is set at a threshold of 40, considered to be the minimum tSNR required to reliably detect effects between conditions in fMRI data (Murphy et al., 2007; Simmons et al., 2010; Wang et al., 2013) and is displayed as a range from 40 (dark blue) to 200 (bright green). Note that the signal reached the minimum threshold throughout the ATL and inferior frontal regions with many ATL subregions reaching a tSNR of 200.

A whole-brain analysis was performed to assess changes in the BOLD signal for our contrasts of interest. An average signal-to-noise map is reported in Figure 2. The map was set at a threshold of 40, which is considered to be the minimum tSNR to detect BOLD differences (Murphy et al., 2007; Wang et al., 2013). The image shows that the acquisition sequence used in this article has ensured minimum BOLD signal within the ATL. A detailed description of the regions significantly activated for each contrast is provided below.

### Conjunction analysis for FAMOUS FACE vs. UNFAMOUS FACE and FAMOUS NAME vs. UNFAMOUS NAME conditions

**Figure 3:**
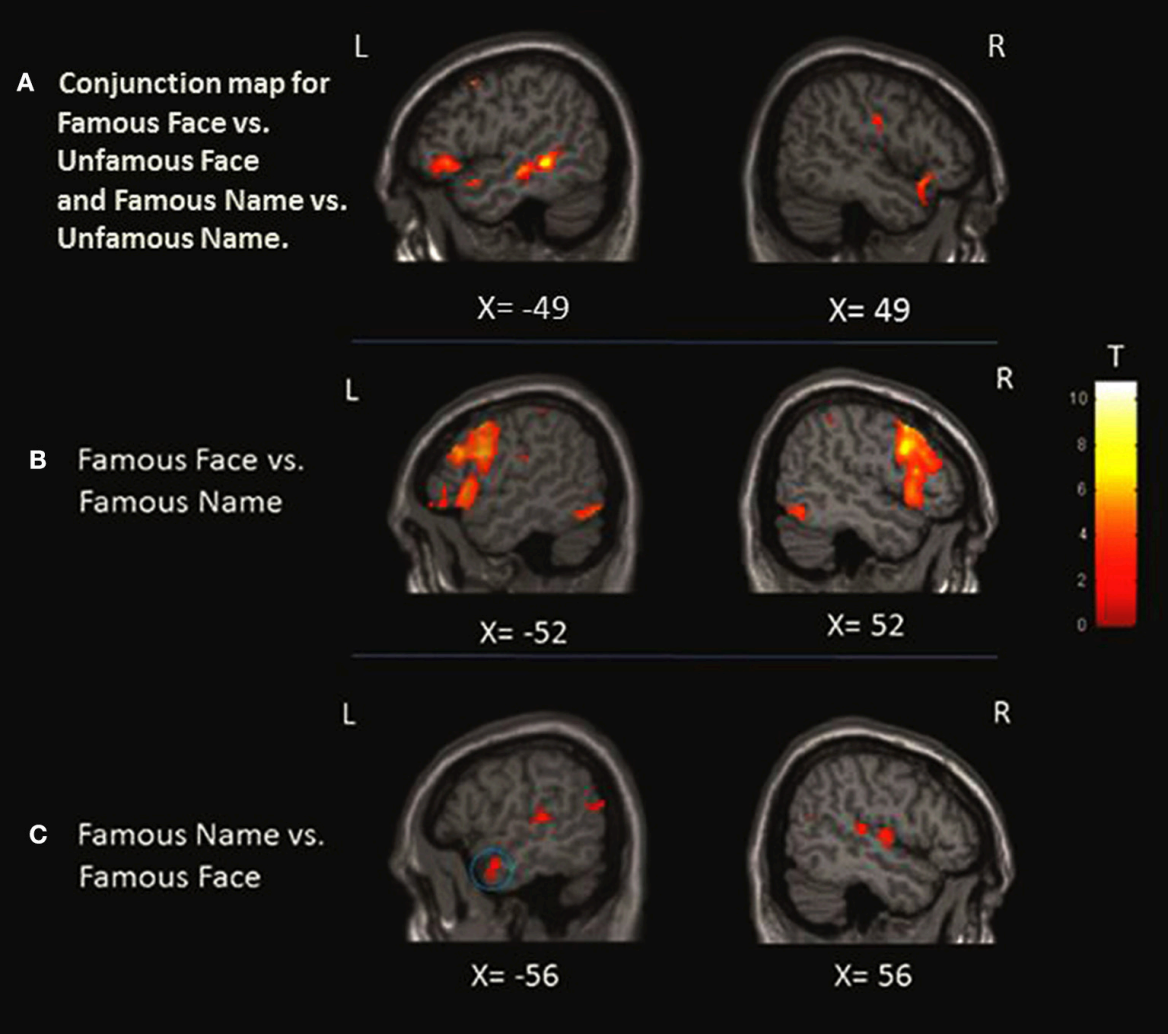
**Localization of the activation in the anterior temporal lobe (ATL)**. **(A)** Conjunction analysis showing regions significantly activated for famous face condition greater than unfamous face and famous name condition greater than unfamous name observed at *x* = −49 and *x* = 49. **(B)** When we compared the faces to names, only left activation was found in the medial portion of the ATL as shown at *X* = −36 and *Z* = −24. **(C)** Left ATL activation at *X* = −56 was observed when we compared the names to faces. The blue circles highlight the specific region where the activation was found at the level of the ATL.

This analysis was performed to identify the common pattern of activation involved in the semantic processing of famous names and faces. The results showed a significant pattern of activation involving the inferior frontal gyrus bilaterally, the anterior cingulate cortex bilaterally, the superior occipital gyrus, the middle temporal gyrus bilaterally, and the temporal pole bilaterally. There was also a significant increase of activation in the right middle frontal gyrus, the left precentral gyrus, the left superior medial frontal gyrus, the right supplementary motor area, the left insula, and the left superior temporal gyrus (Table 4; Figure 3).

**Table 4:** **Conjunction analysis for FAMOUS FACE vs. UNFAMOUS FACE and FAMOUS NAME vs. UNFAMOUS NAME conditions**.

**Anatomical regions**	**Stereotaxic coordinates**			**Z-score**
	***x***	***y***	***z***	
**INFERIOR FRONTAL GYRUS, PARS OPERCULARIS**				
Left	−36	−2	24	4.79
Left	−30	0	32	4.34
Right	40	18	12	3.34
**INFERIOR FRONTAL GYRUS, PARS TRIANGULARIS**				
Left	−52	28	−2	3.81
Right	36	32	10	3.18
Right	30	30	10	3.17
**INFERIOR FRONTAL GYRUS, PARS ORBITALIS**				
Left	−46	18	−10	3.52
Left	−50	36	−8	3.17
Right	48	24	−8	3.47
Right	30	16	12	3.52
**MIDDLE FRONTAL GYRUS**				
Right	34	52	22	3.21
**PRECENTRAL GYRUS**				
Left	−40	2	36	3.68
Left	−34	0	52	3.5
Left	−48	10	50	3.31
**ANTERIOR CINGULATE CORTEX**				
Left	−2	18	26	3.41
Left	−8	40	14	3.34
Right	6	24	24	3.94
Right	12	26	18	3.52
**SUPERIOR MEDIAL FRONTAL GYRUS**				
Left	−2	26	40	3.5
**SUPPLEMENTARY MOTOR AREA**				
Right	10	−6	48	3.49
Insula				
Left	−32	22	−6	3.25
Left	32	18	−10	3.98
**SUPERIOR TEMPORAL GYRUS**				
Left	−56	0	−4	3.14
Left	−40	−38	20	3.11
**MIDDLE TEMPORAL GYRUS**				
Left	−44	−40	−2	4.46
Left	−58	−22	−6	4.3
Right	64	−22	−4	3.5
**TEMPORAL POLE**				
Left	−52	12	−18	3.8
Left	−44	14	−14	3.38
Right	54	18	−14	3.6
**SUPERIOR OCCIPITAL GYRUS**				
Left	−16	−92	14	3.02
Right	20	−90	18	3.92

### FAMOUS FACE vs. FAMOUS NAME conditions

This contrast was performed to assess the perceptual processing of face recognition and semantic retrieval. Within the anterior temporal lobes (ATLs), a cluster of increased activity was observed in the medial portion of the left ATL. No significant differences in activation were observed in the right ATL. Also, the analysis revealed significant increase of activation in the bilateral paracingulate cortices, fronto-insular cortices, supramarginal gyri, and intraparietal sulci, the left superior parietal lobule, the right thalamus, the left premotor cortex, the inferior frontal junction bilaterally, the bilateral fusiform, and occipital cortices (Table 5; Figure 3).

**Table 5:** **Contrast: famous faces vs. famous names**.

**Anatomical regions**	**Stereotaxic coordinates**			**Z-score**
	***x***	***y***	***z***	
**FRONTO-POLAR CORTEX**				
Right	40	62	6	4.33
**PARS TRIANGULARIS**				
Left	−50	28	30	5.06
Left	−42	26	10	4.72
**PARACINGULATE CORTEX**				
Left	−6	18	46	4.71
Right	6	24	42	4.36
**MIDDLE PREFRONTAL CORTEX**				
Right	50	24	42	4.68
**MEDIAL PORTION OF THE ANTERIOR TEMPORAL LOBE**				
Left	−36	24	−24	4.39
**PARS ORBITALIS**				
Left	−46	22	−10	4.50
**FRONTO-INSULAR CORTEX**				
Left	−32	20	2	5.55
Right	30	22	2	5.20
Right	42	24	14	5.31
**PARS OPERCULARIS**				
Right	48	14	6	4.36
**LATERAL PREFRONTAL CORTEX**				
Right	52	14	44	4.99
**INFERIOR FRONTAL JUNCTION**				
Left	−46	12	30	5.44
Right	48	10	30	5.67
**PREMOTOR CORTEX**				
Left	−38	−6	30	5.33
**LATERAL THALAMUS**				
Right	26	−24	0	4.52
**POST-CENTRAL GYRUS/SUPRAMARGINAL GYRUS**				
Left	−46	−38	44	4.33
Right	48	−40	50	4.27
**INTRAPARIETAL SULCUS**				
Left	−24	−50	38	4.47
Right	36	−54	54	4.34
Right	30	−64	40	4.13
**CEREBELLUM**				
Left	−34	−56	−28	4.92
Right	34	−54	−30	5.38
**SUPERIOR PARIETAL LOBULE**				
Left	−28	−62	52	5.61
**FUSIFORM AREA**				
Left	−42	−58	−16	5.86
Left	−40	−68	−14	5.98
Right	38	−80	−12	5.20
**OCCIPITAL CORTEX**				
Left	−40	−84	−8	5.99
Left	−24	−98	6	4.97
Right	24	−98	4	5.80
**PUTAMEN**				
Right	34	4	−2	4.21

### FAMOUS NAME vs. FAMOUS FACE conditions

We observed a significant increase in activation in the lateral portion of the left ATL, and more specifically in the anterior superior temporal gyrus (aSTG). There were no significant differences of activation in the right ATL. Significant activation was found in the posterior superior temporal gyrus bilaterally, the posterior parietal cortex bilaterally, the angular gyrus and the precuneus bilaterally, and the left cuneus and caudate nucleus (Table 6; Figure 3).

**Table 6:** **Contrast: famous names vs. famous faces**.

**Anatomical regions**	**Stereotaxic coordinates**			**Z-score**
	***x***	***y***	***z***	
**ANTERIOR CINGULATE CORTEX**				
Left	−2	32	2	4.40
Right	2	34	12	4.31
Right	8	52	4	4.26
**CAUDATE NUCLEUS**				
Left	−4	20	0	5.04
**ANTERIOR TEMPORAL LOBE**				
Left	−56	0	−20	3.59^*^
**TRANSVERSE GYRUS/POSTERIOR INSULAR CORTEX**				
Left	−42	−34	12	4.55
Right	48	−12	6	3.91
**POSTERIOR SUPERIOR TEMPORAL GYRUS**				
Left	−52	−30	8	3.99
Right	58	−26	10	4.83
**POSTERIOR CINGULATE CORTEX**				
Left	−6	−34	46	5.02
**FUSIFORM CORTEX**				
Left	−32	−44	−6	4.67
**PRECUNEUS**				
Left	−4	−48	48	5.39
Right	4	−44	46	5.20
**CUNEUS**				
Left	−16	−64	22	5.16
**POSTERIOR PARIETAL CORTEX**				
Left	−34	−80	42	4.74
Right	38	−82	34	4.77
**ANGULAR GYRUS**				
Left	−40	−82	36	4.42
Right	48	−74	30	4.69

* *p < 0.001 uncorrected for multiple comparison*.

### Functional connectivity results

**Figure 4:**
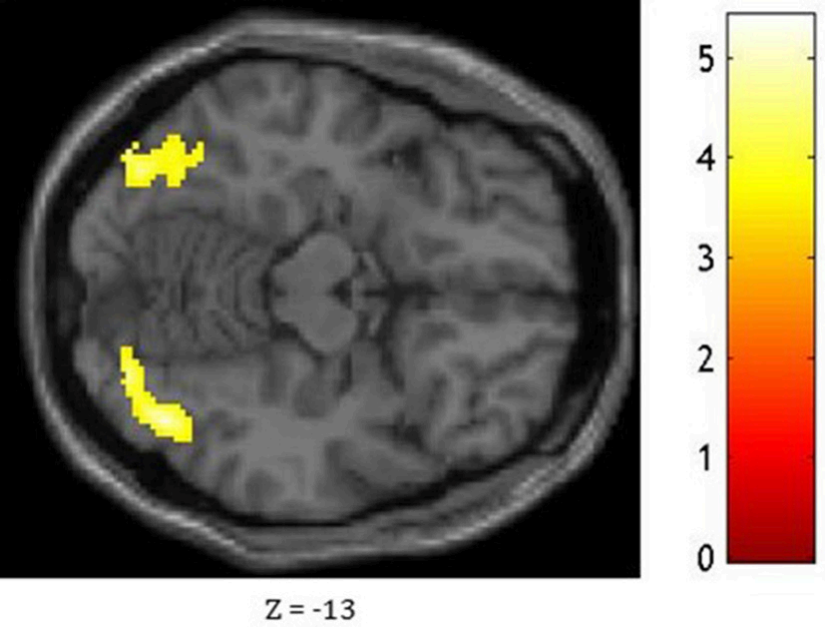
**Statistical significance for the bilateral occipital lobe and fusiform gyrus, the right lingual gyrus, the right inferior temporal gyrus, and the left calcarine fissure while comparing the pattern of FC associated with the medial ATL and to that associated with the anterior STG as shown at ***Z*** = −13**.

Based on fMRI results after contrasting two conditions: Verbal (Famous Name vs. Famous Face) and non-verbal (Famous Face vs. Famous Name), we chose the anterior STG (*X* = −56, *Y* = 0, *Z* = −20) and the medial ATL (*X* = −36, *Y* = 24, *Z* = −24) respectively as regions of interest. A sphere of 8 mm radius based on the coordinates obtained on the previous analysis was used as region of interest (ROI). The maps of functional connectivity (FC) obtained for each ROI were entered in a second level analyses aimed at comparing the differences between the patterns of FC anchored to the medial ATL and the anterior STG. When we compared the pattern of FC associated with the medial ATL to the one associated with the anterior STG, we observed significant differences at the level of the bilateral occipital lobe and fusiform gyrus, the right lingual gyrus, the right inferior temporal gyrus, and the left calcarine fissure (Table 7; Figure 4). We did not observe significant differences when we performed the reverse comparison.

**Table 7:** **Functional connectivity results**.

**Anatomical regions**	**Stereotaxic coordinates**			**Z-score**
	***x***	***y***	***z***	
**MIDDLE OCCIPITAL GYRUS**				
Left	−16	−88	−6	3.91
Left	32	−90	−8	3.96
Right	30	−94	16	3.89
Right	26	−98	10	3.52
**INFERIOR OCCIPITAL GYRUS**				
Left	−24	−92	−2	4.27
Left	−36	−84	10	3.86
Left	−34	−90	−2	3.34
Right	40	−76	−10	4.44
Right	38	−80	−6	4.43
Right	42	−70	−14	4.22
**LINGUAL GYRUS**				
Right	20	−92	−6	3.79
Right	22	−84	−12	3.78
**FUSIFORM GYRUS**				
Left	−38	−76	−12	4.32
Left	−38	−58	−20	4.13
Left	−44	−54	−24	4.05
Left	−40	−82	−14	3.85
Right	32	−70	−22	4.06
Right	30	−74	−20	3.94
Right	28	−82	−14	3.91
Right	30	−78	−16	3.72
**INFERIOR TEMPORAL GYRUS**				
Right	46	−70	−6	3.73
Right	50	−58	−18	3.17
**CALCARINE FISSURE**				
Left	−10	−96	−6	3.48

## Discussion

The present study aimed to investigate the brain network sustaining the semantic processing of famous people. More specifically, the study aimed to map the effect of presentation modality on the pattern of activation associated with the semantic processing of known people, with a particular emphasis on the role of the ATLs. To this aim, we used fMRI with a group of young participants during the execution of a semantic classification task based on the profession of highly recognizable famous people. Two presentation modalities were included in the study: Images (famous faces) and names (famous proper names). We did not observe a right over left lateralized effect in famous person knowledge. Within the ATL, we found greater activation of the left anterior STG for names compared to faces, and of the left medial ATL for faces compared to names. This finding support the models postulating some degree of hemispheric specialization, organized by semantic category (names vs. faces; Damasio et al., 2004; Rice et al., 2015). The analysis showed that various sub-regions within the ATL are differentially implicated in the semantic processing of famous people depending on input modality. The results seem to confirm the results of studies on object semantics showing differential implication of the ATL for verbal and non-verbal material within. More specifically the anterior STG would be more implicated in the semantic processing of names while the medial ATL for images. Nonetheless, we have demonstrated that, compared to the anterior STG, the medial ATL is more functionally connected to the occipital and fusiform regions usually implicated in the visuo-perceptual processing of faces. This pattern of connectivity could explain the specialization of the medial ATL in the semantic processing based on image input.

Identifying known people is a complex and fundamental skill that is necessary for everyday interactions. For this reason, many studies have attempted to identify the brain networks supporting this ability. The majority of functional neuroimaging studies that have aimed to characterize the brain network sustaining the processing of famous people have used a variety of tasks mainly involving stimuli of famous faces (Sergent et al., 1992; Kapur et al., 1995; Gorno-Tempini et al., 1998; Leveroni et al., 2000; Ishai et al., 2005; Brambati et al., 2010). According to these studies, it has been proposed that the neural network sustaining face processing is based on two components: (1) a “core system,” including occipitotemporal regions in the extrastriate visual cortex, mediating visual analysis of faces; and (2) an “extended system,” consisting of regions that are also part of neural systems for other cognitive functions, working in concert with the regions of the “core system” to extract information from faces such as personal identity, name, biographical information, etc… (Haxby et al., 2000). This extended system would include the intraparietal sulcus (spatially directed attention), the auditory cortex (prelexical language perception), the amygdala, the insula, the limbic system (emotion processing, emotion response), and the lateral ATLs (personal identity, name, biographical information). The extended network is justified by the growing evidence that sensory input of one modality can lead to neuronal responses or modified processing in sensory areas of another modality (Besle et al., 2008). For example, a unimodal input (e.g., face) could activate the processing zone of auditory perception, which is the same region for the retrieval of identity information for face recognition. This can be confirmed by the results of the conjunction analysis comparing famous condition to unfamous condition for both modalities, faces and names, showing a bilateral activation of the temporal pole and the anterior cingulate cortex, but a greater activation of the left STG for famous people. Based on a cluster analysis by Woodard et al. (2007) of fMRI BOLD activation during a familiarity task, the cingulate region in addition to the parietal cortex may play a pivotal role in the retrieval of person identity information and familiarity by coordinating multimodal input from a variety of brain regions (Woodard et al., 2007).

Although neuroimaging studies on face processing have greatly increased our knowledge of the organization of the person identity system within the brain, many issues still remain unresolved. One debated issue is whether the input modality for famous people can modulate ATL activation. Two previous functional neuroimaging studies mapped the shared and unique regions of the brain implicated in the processing of faces and proper names of famous people (Gorno-Tempini et al., 1998; Nielson et al., 2010). The two studies reported common regions including the prefrontal cortex, the medial frontal lobe, and the temporo-parietal regions during the processing of famous faces and names compared to unknown stimuli. However, neither of these studies reported significant activation of the ATLs associated with the processing of famous compared to unfamous stimuli. These results are surprising, as they seem to contradict the observations made with neurological patients. Indeed, a great number of studies have shown that patients with damage to the ATLs bilaterally, such as those suffering from svPPA, manifest major impairments in recognizing known people, including friends, relatives, and famous people (Ellis et al., 1989; Evans et al., 1995; Gainotti et al., 2003; Gorno-Tempini et al., 2004; Thompson et al., 2004; Joubert et al., 2006; Lambon Ralph, 2014; Vitali et al., 2015). Two main factors could have contributed to this lack of activation of the ATLs. Firstly, both studies used experimental tasks that could be performed without explicit access to person-specific semantic information: Familiarity judgment (Nielson et al., 2010), and visual judgment task (Gorno-Tempini et al., 1998). Secondly, the study by Nielson and colleagues used fMRI with an axial-oriented acquisition, which was shown to be very sensitive to the potential magnetic susceptibility created by the air-filled sinus and bone near the anterior temporal area. This was shown to attenuate the BOLD signal. Conversely, in our study, we used a specifically designed fMRI acquisition sequence oriented off-axis from the AC-PC line with a smaller voxel size. This sequence was shown to minimize the magnetic susceptibility artifact in the temporal area. Thus, our study was designed to overcome these possible limitations that could have determined the lack of activation in the ATLs in previous reports. Firstly, we used a semantic categorization task based on profession. This is a demanding semantic task requiring explicit access to person-specific semantic information, as previously reported by our group (Brambati et al., 2010). Secondly, we used an fMRI acquisition sequence that has proven to be capable of detecting BOLD signal in the ATLs (see tSNR map, Figure 2; Brambati et al., 2010; Wilson et al., 2012). In order to map the regions involved in the semantic processing of known people, we did a conjunction analysis by comparing the famous items, regardless of the modality of presentation, to unknown items, as done in previous studies (Gorno-Tempini et al., 1998; Nielson et al., 2010). Together with the network of regions previously reported (i.e., the bilateral prefrontal cortex, the temporo-parietal regions, and the medial frontal cortex), the results showed a significant activation of both the left and right ATLs (Brambati et al., 2010). This result indicates that, regardless of the modality of presentation, the semantic processing of famous people is sustained by a bilateral pattern of activation of the ATLs in regions that have shown to be activated during the semantic processing of objects (Binney et al., 2010; Visser et al., 2010; Visser and Lambon Ralph, 2011; Lambon Ralph, 2014). However, some technical limitations such as signal distortion and dropout due to magnetic inhomogeneities are limiting factors in the detection of ventral and polar ATL activation (Binney et al., 2010). Another limitation concerning the choice of baseline contrast for subtraction analysis (pictures or names vs. crosshair fixation, and famous condition vs. unfamous condition) could have been a confounding factor regarding the semantic processing during crosshair fixation or extra effort involved in the perception of unfamous condition.

On the other hand, the comparison between names and faces revealed increased activation in the left anterior STG. Moreover, increased activation was also observed in more posterior regions including the temporal lobe and parietal lobes. These findings are consistent with the model proposing that, during more “verbal” semantic tasks, the semantic system bilaterally distributed in the ATLs would rely more heavily on the left ATL because of its connections with a left-lateralized phonological system (Lambon Ralph et al., 2001) and/or auditory areas (Lambon Ralph, 2014). Consistent with this idea, tasks relying on the language system and involving semantics, such as the naming of known people, often rely on the left ATL (Damasio et al., 2004). A recent electrocorticographical recordings study from epileptic patients has revealed robust electrophysiological responses within the left ATL following the naming of famous individuals (e.g., U.S. Presidents) via pictures or voices (Abel et al., 2015). These results seemed to confirm that when the modality of presentation or the nature of the task requires strong connections with the left lateralized phonological system, the left ATL would be more involved. Again, these results seem to go beyond the nature of our stimuli (famous people). In fact, the region of increased activation that we found in our study is remarkably overlapping with the one observed when they compared object names with pictures (Vandenberghe et al., 1996; Moore and Price, 1999; Visser and Lambon Ralph, 2011; Visser et al., 2012).

Our results seem to be in line with previous findings obtained with object stimuli, highlighting that semantic knowledge would be sustained by the bilateral ATLs. Firstly, a pioneer study by Vandenberghe et al. (1996) reported the presence of a common semantic system for objects, words and pictures extended from the left superior occipital gyrus, through the anterior temporal cortex, and the prefrontal cortex (Vandenberghe et al., 1996). Secondly, a recent meta-analysis of neuroimaging studies on the topic of semantic memory has reported that the type of stimuli (e.g., object, faces, etc.) does not influence the likelihood of ATL activation (Visser et al., 2010). Moreover, spoken words, written words, and picture stimuli were shown to produce overlapping ATL peaks with different levels of activation within the ATL for semantic representation (Spitsyna et al., 2006; Binney et al., 2010; Visser et al., 2010; Visser and Lambon Ralph, 2011; Lambon Ralph, 2014). Furthermore, it has been shown that repetitive TMS applied to the left or right temporal poles equally affects the semantic processing of words and pictures, confirming that both the right and left ATLs support conceptual knowledge, regardless of the modality of stimulus presentation (Pobric et al., 2010).

Our findings seem to provide crucial evidence for the hypothesis that person semantic knowledge may be bilaterally distributed in the ATLs, as previously observed with other types of stimuli such as objects. However, it has been proposed that input modality could modulate ATL activation. In particular, Snowden and colleagues have shown that patients with svPPA with greater left-sided atrophy were better in face recognition compared to famous proper names, while patients with greater right-sided atrophy were better in famous proper names compared to faces (Snowden et al., 2004, 2012). Although this pattern was not consistently replicated across studies, this observation has raised the possibility of a division of labor across the left and right ATLs, due to the dominance of the left hemisphere for language and of the right hemisphere for perceptual pictorial processing (Gainotti, 2007). Recent evidence seems to suggest a differential involvement of different regions within the ATLs and that this could be stronger than any left vs. right laterality effects. More specifically, the superior ATL shows greater activation for auditory and verbal stimuli compared to pictures (Moore and Price, 1999; Visser and Lambon Ralph, 2011; Visser et al., 2012), while ventromedial ATL for pictures relative to words (Visser et al., 2012).

In order to test whether distinct neural circuits underpin the semantic processing of pictures and words, we compared the pattern of activation associated with the semantic processing of famous faces and names and vice versa. When faces were compared to names, we found increased activation in the left inferior prefrontal cortex and the fusiform area, which is consistent with previous findings (Gorno-Tempini et al., 1998; Nielson et al., 2010). The only difference observed at the level of the ATL was located in its medial portion, in regions that were activated in the comparison picture vs. words for object stimuli (Visser and Lambon Ralph, 2011; Visser et al., 2012). No differences were observed in the more inferior temporal regions, as reported in previous studies on objects. This can be due to the fact that the fMRI acquisition sequence could have been less efficient in detecting BOLD signal in inferior ATL. The functional connectivity analysis revealed that, compared to the anterior STG that was more activated for name stimuli, the medial ATL presented stronger functional connectivity with the occipital lobes and fusiform gyri that have a key role in the visuo-perceptual processing of face (and images in general). This connectivity result could support the idea that the relative specialization of the media ATL for image input modality can be determined by functional connectivity with the visuo-perceptual regions.

In conclusion our study provides critical evidence that person-specific semantics is bilaterally sustained by the ATLs. However, differential involvement of ATL regions can be observed with the left aSTG more involved in the processing of names and left medial ATL with pictures, confirming what previously observed for object stimuli. These portions of the ATL are functionally connected to other brain regions, especially the medial ATL that presents strong interactions with the occipital lobe and the furisform gyrus while processing famous faces.

## Author contributions

Study conception and design: MW, SB, SJ, and IR. Acquisition of Data: MW, SB. Analysis and interpretation of data: GC, MW, SB, and JP. Drafting of manuscript: GC, SB. Critical revision: MW, JP, SJ, and IR.

## Funding

SB is supported by Fonds de recherche du Québec - Santé (FRQS) Chercheur Boursier Scholarship (22595). The work is supported by the Natural Sciences and Engineering Research Council of Canada (418630-2012).

### Conflict of interest statement

The authors declare that the research was conducted in the absence of any commercial or financial relationships that could be construed as a potential conflict of interest.
